# Trends in Vaccine Refusal and Acceptance Using Electronic Health Records from a Large Pediatric Hospital Network, 2013–2020: Strategies for Change

**DOI:** 10.3390/vaccines10101688

**Published:** 2022-10-10

**Authors:** Angela K. Shen, Robert W. Grundmeier, Jeremey J. Michel

**Affiliations:** 1 Vaccine Education Center, Children’s Hospital of Philadelphia, Philadelphia, PA 19104, USA; 2 Leonard Davis Institute of Health Economics, University of Pennsylvania, Philadelphia, PA 19104, USA; 3 Department of Medical Bioethics and Health Policy, Perelman School of Medicine, University of Pennsylvania, Philadelphia, PA 19104, USA; 4 Department of Pediatrics, Perelman School of Medicine, University of Pennsylvania, Philadelphia, PA 19146, USA; 5 Center for Pediatric Clinical Effectiveness, Children’s Hospital of Philadelphia, Philadelphia, PA 19146, USA; 6 ECRI Guidelines Trust, ECRI Plymouth Meeting, Philadelphia, PA 19462, USA

**Keywords:** vaccine refusal, vaccine acceptance, vaccine hesitancy

## Abstract

Understanding trends in vaccine refusal is critical to monitor as small declines in vaccination coverage can lead to outbreaks of vaccine-preventable diseases. Using electronic heath record (EHR) data from the Children’s Hospital of Philadelphia’s 31 outpatient primary care sites, we created a cohort of 403,448 children less than age 20 years who received at least one visit from 1 January 2013 through 31 December 2020. The sample represented 1,449,061 annualized patient and 181,131 annualized preventive vaccination visits per year. We characterized trends in vaccine refusal and acceptance using a repeated cross-sectional observational analysis of electronic health records (EHR) data using a single annual merged observation measure for patients seen multiple times for preventive healthcare within a calendar year. Refusals were identified for 212,900 annualized patient-visit year observations, which represented 14.6% of annualized patient-visit year observations and 25.1% of annualized vaccine patient-year observations. The odds of having a refusal marker were significantly increased in patients seen in suburban practices (aOR [CI]: 2.35 [2.30–2.40, *p* < 0.001]), in patients with increased age 11–17 years (aOR [CI]: 3.85 [3.79–3.91], *p* < 0.001), and those eligible for the VFC program (aOR [CI]: 1.10 [1.08–1.11]. Parental refusal (61.0%) and provider decisions (32.0%) were the most common documented in progress notes for not administering vaccines, whereas contraindications (2.5%) and supply issues (1.8%) were the least common. When offered, vaccine acceptance increased for human papillomavirus, hepatitis B, measles-mumps-rubella-containing and varicella-containing vaccines and decreased for hepatitis A and meningococcal vaccines. Repeated offering of vaccines was central to increasing acceptance, in part due to increased opportunities to address specific concerns.

## 1. Background

Vaccine hesitancy—the delay in acceptance or refusal of vaccination despite availability of vaccine supply and services—is an urgent public health issue as evidenced in part by recent, large measles outbreaks in the United States and current challenges to achieving the COVID-19 vaccine coverage necessary to slow the spread of COVID-19 [1,2,3,4]. Although childhood vaccination rates are generally high in the United States [5], some parents are generally hesitant to vaccinate and others are doubtful about specific Advisory Committee on Immunization Practices (ACIP) routinely-recommended vaccines. For example, approximately 26% of parents report hesitancy related to influenza vaccine and about 23% for human papillomavirus vaccine (HPV) vaccine. Concerns are often centered around vaccine side effects, disease severity, and vaccine effectiveness [6,7]. Similar concerns have been expressed about COVID-19 vaccines [8,9,10].

Vaccine hesitancy is complex, varying over time, context, and vaccine [11]. Though some parents may opt to vaccinate their children in spite of minor concerns, others choose to delay all vaccines or only accept certain vaccines [12,13]. Even parents with minor concerns often have questions and may not know how to get their questions answered or who to trust [14]. As such, the nature of the provider–patient family relationship (good, neutral, non-existent) is critical [15]. A trusting provider–patient relationship lends itself to an environment supportive of vaccine conversations and positions the provider to influence parental positions on vaccination [12,13].

Because individual vaccine decisions affect the cumulative health of a community, adequate vaccine coverage and timely vaccine acceptance are critical to understand. Experience has demonstrated that even small declines in vaccination coverage can result in pockets of susceptible individuals in communities, leading to substantial public health and economic consequences [16]. As such, public health officials and large hospital networks, like the Children’s Hospital of Philadelphia (CHOP), have sought to not only monitor local vaccination environments, but to also target specific vaccines with low coverage rates (e.g., HPV vaccine) [17]. Focused efforts to identify children due for recommended vaccines and implementation of strategies to catch-up on missed vaccines have been demonstrated to increase coverage levels [18,19]. To understand trends of vaccine refusal over time and to identify whether trends varied by vaccine, we used CHOP’s electronic health record (EHR) to evaluate patient- and vaccine-level trends from 2013 to 2020.

## 2. Methods

Data Source and Study Subjects: The CHOP network is comprised of 31 primary care sites that serve over one million patients annually. The network includes six urban sites, of which three are “academic” where resident and fellow training occurs and 25 suburban primary care sites (22 in Pennsylvania and three in New Jersey), where all providers have completed training [20]. In addition to having providers in training, academic sites have a higher proportion of patients eligible for the Vaccines for Children Program (VFC), the federal safety net that ensures eligible children who are uninsured, American Indian or Alaska native, or Medicaid-eligible have public insurance coverage for vaccination services [21].

The study was completed using EHR data (Epic Systems, Inc., Verona, WI, USA) from all CHOP network sites. Data represented children through to 19 years of age who received care between 1 January 2013, and 31 December 2020. The cohort included 403,488 unique patients seen at least once during the study period and represented 1,449,061 annualized patient visits.

Variables and Outcomes: We extracted patient-level demographic variables (gender, race, ethnicity), visit-level data [year of service, age at time of visit (0–11 months, 1–10 years, 11–17 years, 18–19 years), insurance/VFC eligibility status, clinic location, diagnosis codes], and vaccination-level data (documentation of vaccine administration, contraindications, refusal, and reasons for refusal). Annualized patient-year observation cohorts were created for each calendar year (2013–2020) by calculating the number of patients multiplied by the number of visits per year. Demographics were obtained from information listed in the first preventive care visit of each year. For visit-level and vaccination-level data, we aggregated all EHR system generated prompts for ACIP-routinely recommended vaccines [22] to determine when vaccinations were due and to identify vaccine acceptance and vaccine refusal markers over each year of observation for each patient. Vaccine acceptance was defined as accepted (i.e., administered vaccination) over the number of times offered.

Vaccine refusal was identified using four EHR markers:

(1) Marker 1: Clinician documentation in the progress note. This visit-level marker comprises a dropdown list of reasons that clinicians can select when any vaccine is refused. Choices include parent or patient refusal, contraindications, scheduling considerations, incomplete records, and vaccine availability.
(2) Marker 2: Visit diagnosis. This visit-level marker relies on vaccine refusal diagnosis codes derived from the International Classification of Diseases (ICD) diagnosis codes (ICD-9 codes: V64.00, V64.05, V64.09, V64.06 or V64.07 and ICD-10 codes: Z28.1, Z28.2x, Z28.82, Z28.83, Z28.89, Z28.9).
(3) Marker 3. Problem list entry. Using ICD codes from Marker 2, vaccine refusal can be documented in a section of the EHR called the “problem list”. This section helps providers track active and resolved chronic health conditions that may impact current or future patient care decisions [23]. This marker was counted as a patient-level marker and is the only marker that disables clinical decision support to the provider.
(4) Marker 4. Vaccine refusal letter. Some patient records include a signed vaccine refusal letter which discusses the benefits of vaccination. Parents sign that they have reviewed the letter and wish to decline vaccinations for their child.

Vaccine refusal is defined as one or more of these markers in a patient record and is not specific to a specific antigen. Patient refusal status could change over time and can be independent of vaccination intent.

Analysis: We performed a repeated cross-sectional observational analysis of utilization patterns of EHR refusal markers by year and over time. Our primary unit of analysis was person-years, with data from patients who were seen for multiple visits within a calendar year merged into a single observation, the “annualized patient visit”. Vaccine refusal and acceptance was calculated across annual cohorts of patients based on demographic variables (practice type, gender, race, ethnicity, age, and payor type) and vaccine (i.e., antigen). Confidence intervals around proportions were calculated using the prop.test() function in R.

We analyzed the relationship between individual study variables of gender, race, ethnicity, VFC-eligibility (as a marker for insurance status), practice group location, and age at visit with the main outcome of documentation of refusal markers. Significance of each variable was assessed using chi-square, and all significant variables were evaluated using multivariable logistic regression analysis using the glm() function in R.

For adolescents only, we calculated vaccination coverage by the Healthcare Effectiveness Data and Information Set^®^(HEDIS) Immunizations for Adolescents (IMA) quality measure [one dose of meningococcal vaccine, one tetanus, diphtheria, acellular pertussis (Tdap) or tetanus (TD) vaccine and the complete human papillomavirus vaccine (HPV) series by an adolescents’ 13th birthday (2 or 3 doses depending on year of analysis). HPV 2-dose recommendations for those younger than 15 years of age were published in 2016 [24,25,26]. All analyses were performed using RStudio version 1.0.136 (RStudio, PBC, Boston, MA, USA) [27]. This study was considered exempt by the CHOP Institutional Review Board.

## 3. Results

A total of 403,488 unique patients were seen at a preventive care visit at least once during the study period (Table 1). Most patients (67.6%) received preventive care at suburban sites (n = 272,895), followed by 21.6% at academic (n = 86,952) and 10.8% at urban non-academic sites (n = 43,461) (Table 1).

Markers of refusal were present at 212,900 annualized patient visits from all 1,449,061 annualized patient visit years of observation. This accounted for 14.6% annualized visits in total and 25.1% of 847,890 annualized patient visits when a vaccine was due (Table 2).

Visits with a refusal marker increased from 13.1% of all annualized visits in 2013 to a peak of 17.3% in 2019, corresponding to an increase from 21.4% to 28.5% of annualized visits when a vaccine was due (Table 2). Suburban practices in general had the highest refusal rates, reaching 20.0% of all annualized visits in 2019. Refusal rates were highest among children aged 11–17 years and among children who were not eligible for the VFC program (i.e., privately insured). Refusals declined in 2020 across all sites and most demographic variables.

In the multivariable model, the odds of having a documented refusal marker were significantly increased in patients seen in suburban practices (aOR [CI]: 2.35 [2.30–2.40, *p* < 0.001]), in patients with increased age 11–17 years (aOR [CI]: 3.85 [3.79–3.91], *p* < 0.001), and those eligible for the VFC program (aOR [CI]: 1.10 [1.08–1.11] (Table 3). The odds of having a refusal marker were significantly lower for non-females (aOR [CI]: 0.95 [0.94–0.96], in all race categories, and in Hispanic or Latino patients (aOR [CI]: 0.77 [0.75–0.79], *p* < 0.001) (Table 3).

About half of patients with refusal markers (40.3–50.1%) received at least one vaccination (Table 4) compared with a majority of patients without refusal markers (88.2–91.0%) among annualized visits where at least one vaccine was due (data not shown). Across all study years, clinical documentation (refusal marker 1) was most utilized (83.0%) compared with other refusal markers measured (Table 4). A total of 13% of all annualized patient visits included multiple markers. The most common combination of markers (30.7%) was clinical documentation (marker 1) and visit diagnosis (marker 2). For 2.9% (n = 11,899) of those patients with a “problem list” refusal indicator (marker 3), the entry was ultimately removed. The average duration of the entry was 2.37 years (or 832 days). The median age at “problem list” entry removal was 4.5 years (inter quartile range: 2.0–11.8 years).

Vaccine acceptance upon offer changed over time and generally increased for most vaccine antigens [vaccines containing human papilloma virus (HPV), hepatitis B, measles-mumps-rubella (MMR), polio, tetanus and varicella antigens], and generally decreased for hepatitis A, meningococcal, and pneumococcal antigens. Acceptance of HPV vaccine, a vaccination indicated at adolescence (typically at age 11–12 years) was lowest (average 44.4% across years), followed by varicella (78.2%), hepatitis A (79.5%), and hepatitis B (79.8%) containing vaccines (Table 5).

Vaccine coverage rates among adolescents as measured by the HEDIS criteria increased from 41.8% in 2013 to 61.8% in 2020 with the lowest vaccination coverage in suburban sites and the highest in urban academic sites (Table 6). This increase was driven largely by improvements in vaccination rates in the suburban sites, which increased from 36.6% to 57.0% across the study period.

Of the 289,622 annualized visits with “some” or “no” vaccinations given associated with marker 1 (mean of 36,184 annual visits), parental or patient refusal was most often the reason for not vaccinating (60.1%), followed by other clinician or practice related reasons (31.9%), incomplete patient history (4.3%) contraindications to vaccination (2.5%), and vaccine supply shortage (1.8%). 

## 4. Discussion

The aim of this study was to use EHR records to examine trends in vaccine refusal and acceptance in a large outpatient pediatric network over a period of eight years (2013–2020). The study cohort included over 400,000 unique patients and almost 1.5 million annualized patient visits. The overall refusal rate was >14% of annualized patient-visit-year observations and 25.1% of annualized vaccine patient-year observations. Refusal rates increased from 13.1% of annualized visits in 2013 to a peak of 17.3% in 2019. Refusal rates were higher in suburban than in urban sites, highest in children aged 11–17 years and those privately insured. Trends in vaccine acceptance and refusal varied by antigen and enhance the existing literature [1,2,3,4,28,29]. Vaccination coverage in adolescents increased during the study period, a promising finding given the concerted effort to increase HPV vaccination nationwide [30]. Parental or patient refusal was the most common reason for not vaccinating (60.1%).

These results empirically demonstrate continued parental concerns for at least one ACIP-routinely recommended vaccine and though not documented in this study, concerns differ among antigens [28,29]. Providers, as a trusted source of medical information and vaccine advice, should continue to offer vaccines that have been refused, recognizing that over time parents may change their mind [28,29]. Sometimes parents and families need more time and multiple touchpoints with trusted sources to inform their decision-making. Reasons that influence decision-making and subsequent behavior could be many (e.g., provider presentation and offering of vaccinations, social changes, and new information).

To raise vaccination coverage for HPV, CHOP engaged in a quality improvement effort focused on engaging providers and prioritizing HPV vaccination through clinical-focused decision support, EHR-based alerts, transparent data reporting, on-line education sessions, and standing orders, as well as shifting initiation of vaccination earlier to nine years of age [17]. Other strategies anchored in family-focused decision support (e.g., automated reminder phone calls, education support), vaccine champions, and automation and standardization of QI efforts have also proven effective in raising coverage not only in pediatric but also adult efforts to increase vaccination coverage of recommended vaccines like HPV closer to national goals [30,31,32,33,34]. These strategies help support provider recommendations and engagements with families, which are central to increasing vaccination coverage rates.

However, there is also ambivalence in the patient population, as acceptance of some vaccines—hepatitis A, pneumococcal and meningococcal—have decreased. Other vaccines most frequently denied by parents for children include the hepatitis B, rotavirus, MMR, varicella, pneumococcal, and polio vaccines, with uptake of influenza and HPV vaccines remaining suboptimal as well [35,36]. Much of the existing literature have cited common concerns for hesitancy: lack of perceived need, vaccine safety, distrust in healthcare providers and the government, perceived lack of involvement in the decision-making process, immune system overload, religious objections, and lack of adequate time and resource to support vaccine conversations [28,29,35].

Prevalence of vaccine refusal markers also increased more rapidly in our academic urban sites that serve a higher proportion of VFC-eligible children, which suggest vaccine refusal patterns in urban settings may now be mirroring those more typically noted in suburban settings [37]. Our findings echo other studies on vaccine hesitancy and reinforce the notion that confidence in vaccines is nuanced and informed by many factors, which leads to confidence in some vaccines but not others [1,2,3,4,6,7,8,28,29].

This study was unique in evaluating the clinician documentation of vaccine refusal during routine care. Documentation was collected routinely and repeatedly in our primary care network throughout our study period and makes our analyses unique compared to prior studies of vaccine hesitancy. However, these population-level data were not analyzed at the individual patient level, limiting the ability to identify risk factors and predictors for refusal or acceptance. One interesting finding, and its potential implications, that is worth noting is the removal of “problem list” entries which occurred around four years of age (Table 4), suggestive of the importance of preschool or kindergarten-based vaccine requirements. Although this study cannot prove causality, it provides hypothesis-generating evidence to further evaluate potential differences among variables such as setting and insurance status.

In light of the potential for weakening of all vaccine mandates as a result of pushback against COVID-19-based mandates, exploring parental decision-making and views on vaccines can help inform policies and practices around vaccination [38,39]. Toward how to better inform the provider-patient conversation, facilitating detailed documentation of specific concerns about hesitancy in the EHR may be worth exploring as the use of clinical documentation versus other refusal markers such as the refusal letter is relatively easy.

Our study faced some notable limitations. First, the definition of refusal used in this study was based on specific elements of the EHR that may not capture nuanced reasons for vaccine refusal. Furthermore, refusal in our data did not specify whether refusal was to specific antigens or all antigens. Our markers relied on clinical documentation, and it is likely that some clinicians were inconsistent in documenting refusal, which may lead to an under reporting of rates.

Second, the data were not analyzed by patient, but rather aggregated for analysis. In addition, the same cohort of individuals was not followed over time. However, these estimates are still useful for understanding changes to vaccine refusal over time.

An additional limitation of this study is the impact of the COVID-19 pandemic (2020–2022) on perceptions of risk and attitudes about vaccines and vaccinations and the possible selection bias in that children coming to office visits since the COVID-19 pandemic (Spring 2020) may not be the same children who came in the prior year (i.e., parents more favorably disposed to health care or a group less concerned about infectious disease and thus, less fearful about coming to the office).

Finally, our study was conducted in a single network of primary care practices, which may limit the generalizability of our results. However, there are diverse populations and practice cultures represented across the 31 practices in our network, and our analysis of trends in vaccine refusal may generalize to other health systems with similar demographics.

## 5. Conclusions

Although vaccine refusal is complex and based on a variety of factors, repeated offering of vaccines at multiple visits may increase vaccine acceptance and, therefore, vaccination coverage. Health systems should ensure that multiple opportunities exist for providers and patients to have vaccine conversations to both build upon and leverage the trust common to this relationship.

## Figures and Tables

**Table 1:** Baseline Sociodemographics of Study Cohort (N = 403,488), 2013–2020.

	No. (%)			
Patient Demographics, Children’s Hospital of Philadelphia Primary Care Network	Urban Academic N = 86,952	Urban Non-Academic N = 43,641	Suburban N = 272,895	All Sites N = 403,488
Gender				
Female	42,815 (49.2)	21,369 (49.0)	133,274 (48.8)	197,458 (48.9)
Not Female	44,137 (50.8)	22,272 (51.0)	139,621 (51.2)	206,030 (51.1)
Race				
American Indian, Alaska Native	64 (0.1)	42 (0.1)	257 (0.1)	363 (0.1)
Asian, Indian, Native Hawaiian, Other Pacific Islander	3852 (4.4)	1938 (4.4)	13,030 (4.8)	18,820 (4.7)
Black, African American	63,819 (73.4)	18,295 (41.9)	31,208 (11.4)	113,322 (28.1)
White	8141 (9.4)	17,588 (40.3)	179,219 (65.7)	204,948 (50.8)
Multiple	1995 (2.3)	1593 (3.7)	7945 (2.9)	11,533 (2.9)
Unknown	9081 (10.4)	4185 (9.6)	41,236 (15.1)	54,502 (13.5)
Ethnicity				
Not Hispanic or Latino	79,972 (92.0)	40,300 (92.3)	249,276 (91.3)	369,548 (91.6)
Hispanic or Latino	6601 (7.6)	2891 (6.6)	21,250 (7.8)	30,742 (7.6)
Unknown	379 (0.4)	450 (1.0)	2369 (0.9)	3198 (0.8)
Vaccines for Children (VFC) ^a,b^ Eligibility				
VFC Eligible	72,375 (83.2)	15,848 (36.3)	89,838 (32.9)	178,061 (44.1)
Non-VFC Eligible	14,577 (16.8)	27,793 (63.7)	183,057 (67.1)	225,427 (55.9)
Age at First Visit				
Median (IQR)	3.4 (0.0–9.3)	2.9 (0.0–9.3)	4.0 (0.0–9.8)	3.8 (0.0–9.6)
Age at Most Recent Visit				
Median (IQR)	9.4 (4.4–15.0)	9.5 (4.0–16.1)	10.6 (5.0–16.3)	10.2 (4.7–16.1)
Years of Patient Observations				
Median (IQR)	4.6 (2.0–7.2)	5.0 (2.2–8.0)	5.1 (2.4–8.0)	5.0 (2.3–7.8)

^a^ The Vaccines for Children (VFC) Program is a federally funded program that provides vaccines at no cost to eligible children. Eligible children include those who are American Indian/Alaska Native, Medicaid-eligible, uninsured, and underinsured. Underinsured children are eligible to receive VFC vaccine only through a Federally Qualified Health Center (FQHC), Rural Health Clinic (RHC), or under an approved deputization agreement. ^b^ Urban academic sites have a higher proportion of VFC patients and serve as a training site for CHOP physicians.

**Table 2:** Demographics and Markers of Vaccine Refusal of Annualized Preventive Patient-Year Visits in the Study Cohort, 2013–2020 (N = 1,449,061).

	No. (%)								
	All Years	2013	2014	2015	2016	2017	2018	2019	2020 ^c^
All Visits	212,900/1,449,061 (14.7)	22,717/172,931 (13.1)	24,874/181,310 (13.7)	25,001/178,948 (14.0)	25,574/185,902 (13.8)	27,457/188,242 (14.6)	29,853/188,171 (15.9)	31,261/180,192 (17.3)	26,163/173,365 (15.1)
All visits, vaccines offered	212,900/847,890 (25.1)	22,717/105,819 (21.5)	24,874/105,788 (23.5)	25,001/102,470 (24.4)	25,574/103,541 (24.7)	27,457/104,031 (26.4)	29,853/106,435 (28.0)	31,261/109,640 (28.5)	26,163/110,166 (23.7)
All visits, vaccines administered	97,388/666,741 (14.6)	10,333/84,107 (12.3)	11,926/84,072 (14.2)	11,800/81,208 (14.5)	12,100/82,286 (14.7)	12,761/80,345 (15.9)	14,957/83,664 (17.9)	12,946/84,073 (15.4)	10,565/86,986 (12.1)
Practice type									
Urban ^a^ Academic	17,612/274,437 (6.4)	1518/32,446 (4.7)	1717/36,169 (4.7)	1690/35,111 (4.8)	1746/35,982 (4.9)	2025/37,334 (5.4)	3064/35,330 (8.7)	3198/33,094 (9.7)	2654/28,971 (9.2)
Urban Non-Academic	16,618/155,590 (10.7)	2127/19,742 (10.8)	2049/19,302 (10.6)	1971/19,378 (10.2)	1882/20,115 (9.4)	1951/19,769 (9.9)	2132/20,520 (10.4)	2457/19,190 (12.8)	2049/17,574 (11.7)
Suburban	178,670/1,019,034 (17.5)	19,072/120,743 (15.8)	21,108/125,839 (16.8)	21,340/124,459 (17.1)	21,946/129,805 (16.9)	23,481/131,139 (17.9)	24,657/132,321 (18.6)	25,606/127,908 (20.0)	21,460/126,820 (16.9)
Gender									
Female	103,693/706,872 (14.7)	10,680/84,513 (12.6)	11,983/88,560 (13.5)	12,127/87,208 (13.9)	12,406/90,821 (13.7)	13,550/91,721 (14.8)	14,751/91,590 (16.1)	15,294/87,711 (17.4)	12,902/84,748 (15.2)
Not Female	109,207/742,189 (14.7)	12,037/88,418 (13.6)	12,891/92,750 (13.9)	12,874/91,740 (14.0)	13,168/95,081 (13.8)	13,907/96,521 (14.4)	15,102/96,581 (15.6)	15,967/92,481 (17.3)	13,261/88,617 (15.0)
Race									
American Indian, Alaska Native	159/1268 (12.5)	23/152 (15.1)	23/171 (13.5)	14/157 (8.9)	19/153 (12.4)	19/171 (11.1)	21/174 (12.1)	23/150 (15.3)	17/140 (12.1)
Asian, Native Hawaiian, Other Pacific Islander	7085/65,634 (10.8)	650/6347 (10.2)	787/7167 (11.0)	827/7608 (10.9)	875/8397 (10.4)	968/8947 (10.8)	1048/9261 (11.3)	1089/9015 (12.1)	841/8892 (9.5)
Black, African American	36,566/371,885 (9.8)	3508/46,197 (7.6)	3856/49,769 (7.7)	3858/48,213 (8.0)	3845/48,839 (7.9)	4447/49,008 (9.1)	5632/46,962 (12.0)	5990/43,769 (13.7)	5430/39,128 (13.9)
White	139,412/787,081 (17.7)	15,792/96,729 (16.3)	17,228/99,101 (17.4)	17,288/97,557 (17.7)	17,506/100,889 (17.4)	18,271/100,762 (18.1)	18,667/100,982 (18.5)	19,103/96,604 (19.8)	15,557/94,457 (16.5)
Multiple	4287/40,118 (10.7)	257/3183 (8.1)	356/3825 (9.3)	355/4204 (8.4)	476/4842 (9.8)	573/5466 (10.5)	723/5883 (12.3)	821/6184 (13.3)	726/6531 (11.1)
Unknown	25,391/183,075 (13.9)	2487/20,323 (12.2)	2624/21,277 (12.3)	2659/21,209 (12.5)	2853/22,782 (12.5)	3179/23,888 (13.3)	3762/24,909 (15.1)	4235/24,470 (17.3)	3592/24,217 (14.8)
Ethnicity									
Not Hispanic or Latino	200,096/1,333,757 (15.0)	21,672/161,562 (13.4)	23,669/168,952 (14.0)	23,734/165,761 (14.3)	24,141/171,460 (14.1)	25,759/172,844 (14.9)	27,835/172,025 (16.2)	29,055/164,218 (17.7)	24,231/156,935 (15.4)
Hispanic or Latino	11,349/106,000 (10.7)	974/10,756 (9.1)	1099/11,616 (9.5)	1157/12,357 (9.4)	1287/13,423 (9.6)	1508/14,179 (10.6)	1771/14,710 (12.0)	1905/14,356 (13.3)	1648/14,603 (11.3)
Unknown	1455/9304 (15.6)	71/613 (11.6)	106/742 (14.3)	110/830 (13.3)	146/1019 (14.3)	190/1219 (15.6)	247/1436 (17.2)	301/1618 (18.6)	284/1827 (15.5)
Age group									
0–11 months	24,322/203,432 (12.0)	2204/22,858 (9.6)	2458/24,304 (10.1)	2449/25,086 (9.8)	2700/24,927 (10.8)	3026/25,605 (11.8)	4395/26,582 (16.5)	3882/26,872 (14.4)	3208/27,198 (11.8)
1–10 years	49,133/767,776 (6.4)	4106/93,533 (4.4)	4455/97,034 (4.6)	4384/94,480 (4.6)	4692/98,447 (4.8)	5032/99,615 (5.1)	5756/99,005 (5.8)	10,288/95,496 (10.8)	10,420/90,166 (11.6)
11–17 years	132,050/438,044 (30.1)	15,674/52,136 (30.1)	17,238/55,223 (31.2)	17,477/54,592 (32.0)	17,412/57,265 (30.4)	18,120/57,378 (31.6)	18,440/57,166 (32.3)	16,024/53,081 (30.2)	11,665/51,203 (22.8)
18–19 years	7395/39,809 (18.6)	733/4404 (16.6)	723/4749 (15.2)	691/4790 (14.4)	770/5263 (14.6)	1279/5644 (22.7)	1262/5418 (23.3)	1067/4743 (22.5)	870/4798 (18.1)
Vaccines for Children (VFC) Eligibility ^b^									
VFC eligible	46,570/447,934 (10.4)	3600/49,785 (7.2)	4349/55,320 (7.9)	4756/56,136 (8.5)	5142/59,000 (8.7)	5951/60,005 (9.9)	7570/59,005 (12.8)	8042/56,001 (14.4)	7160/52,682 (13.6)
Not VFC eligible	166,330/1,001,127 (16.6)	19,117/123,146 (15.5)	20,525/125,990 (16.3)	20,245/122,812 (16.5)	20,432/126,902 (16.1)	21,506/128,237 (16.8)	22,283/129,166 (17.3)	23,219/124,191 (18.7)	19,003/120,683 (15.7)

^a^ Urban academic sites have a higher proportion of VFC patients and serve as a training site for physicians. ^b^ The Vaccines for Children (VFC) Program is a federally funded program that provides vaccine at no cost to eligible children. Eligible children include those who are American Indian/Alaska Native, Medicaid-eligible, uninsured, and underinsured. Underinsured children are eligible to receive VFC vaccine only through a Federally Qualified Health Center (FQHC), Rural Health Clinic (RHC), or under an approved deputization agreement. ^c^ 2020 was impacted by the COVID-19 pandemic. During this year, CHOP prioritized preventive care visits where vaccinations were indicated according to the Advisory Committee on Immunization Practices (i.e., at well child visits for those <2 years, 4–5 years, and 11 years of age).

**Table 3:** Multivariable logistic regression results for documentation of at least one refusal marker when vaccination was indicated (n = 847,890).

Variables	Adjusted Odds Ratio (aOR)	[95% Confidence Interval]		*p*-Value
Patient Age ^a^ (Reference: Under Age One Year)				
1–10 years	1.33	1.30	1.35	<0.001 *
11–17 years	3.85	3.79	3.91	<0.001 *
18–19 years	3.40	3.29	3.51	<0.001 *
Gender (Reference: Female)				
Not Female	0.95	0.94	0.96	<0.001 *
Race (Reference: White)				
American Indian or Alaska Native	0.72	0.60	0.86	<0.001 *
Asian, Indian, Native Hawaiian or Other Pacific Islander	0.69	0.67	0.71	<0.001 *
Black or African American	0.81	0.80	0.82	<0.001 *
Other or Unknown	0.92	0.91	0.94	<0.001 *
Multiple	0.85	0.82	0.88	<0.001 *
Ethnicity (Reference: Not Hispanic or Latino)				
Hispanic or Latino	0.77	0.75	0.79	<0.001 *
Refused or Unknown	1.19	1.12	1.27	<0.001 *
Practice Location (Reference: Urban Academic) ^b^				
Urban Non-Academic	1.42	1.39	1.46	<0.001 *
Suburban	2.35	2.30	2.40	<0.001 *
Vaccines for Children (VFC) ^c^, Eligible (Reference: No)				
Yes	1.10	1.08	1.11	<0.001 *

* Denotes significant *p*-value (α = 0.05). ^a^ 2020 was impacted by the COVID-19 pandemic. During this year, CHOP prioritized preventive care visits where vaccinations were indicated according to the Advisory Committee on Immunization Practices (i.e., at well child visits for those <2 years, 4–5 years, and 11 years of age). ^b^ Urban academic sites have a higher proportion of VFC patients and serve as a training site for physicians. ^c^ The Vaccines for Children (VFC) Program is a federally funded program that provides vaccine at no cost to eligible children. Eligible children include those who are American Indian/Alaska Native, Medicaid-eligible, uninsured, and underinsured. Underinsured children are eligible to receive VFC vaccine only through a Federally Qualified Health Center (FQHC), Rural Health Clinic (RHC), or under an approved deputization agreement.

**Table 4:** Receipt of Any Vaccines by Patients with Refusal Markers and Distribution of Refusal Markers in the EHR by Marker Type, 2013–2020 (N = 212,900).

	Annualized Visits with Refusal Markers N	Marker 1: Clinical DocumentationN (%)	Marker 2: Visit Diagnosis N (%)	Marker 3: Problem List Entry ^a^ N (%)	Marker 4: Refusal Letter N (%)	Multiple Markers ^b^ N (%)	Any Vaccine Given with Refusal Marker Present N (%)
Year							
2013	22,717	19,118 (84.2)	2502 (11.0)	2433 (10.7)	1190 (5.2)	2413 (10.6)	10,333 (45.4)
2014	24,874	21,101 (84.8)	2865 (11.5)	2484 (10.0)	1538 (6.2)	2962 (11.9)	11,926 (47.9)
2015	25,001	21,161 (84.6)	2749 (11.0)	2358 (9.4)	1703 (6.8)	2833 (11.3)	11,800 (47.2)
2016	25,574	21,367 (83.5)	3473 (13.6)	2708 (10.6)	1982 (7.8)	3615 (14.1)	12,100 (47.3)
2017	27,457	23,296 (84.8)	3838 (14.0)	2577 (9.4)	2185 (8.0)	4146 (15.1)	12,761 (46.5)
2018	29,853	23,851 (79.9)	3887 (13.0)	2319 (7.8)	2341 (7.8)	4180 (14.0)	14,957 (50.1)
2019	31,261	24,911 (79.7)	3797 (12.1)	1663 (5.3)	2573 (8.2)	4062 (13.0)	12,946 (41.4)
2020	26,163	21,811 (83.4)	3553 (13.6)	810 (3.1)	2530 (9.7)	3513 (13.4)	10,565 (40.4)
All Years	212,900	176,616 (83.0)	26,664 (12.5)	17,352 (8.2)	16,042 (7.5)	27,724 (13.0)	97,388 (45.7)

^a^ For 2.9% (n = 11,899) of those patients with Marker 3, the refusal diagnosis is ultimately removed. Average duration of this marker is 2.27 years (or 832 days) and the age at which this marker is removed is 4.54 years (Inter Quartile Range 2.0–11.8). ^b^ Includes any combination of at least two markers at an annualized patient visit. Combination of Markers 1 and 2 are most common (30.7%). NB: Markers 1 and 2 are visit specific; Marker 3 is linked to the patient chart, but removable; and Marker 4 is not removable once added to the patient chart.

**Table 5:** Vaccine Acceptance Upon Provider Recommendation by Antigen, 2013–2020.

	No. (%)								
Antigen	All Years	2013	2014	2015	2016	2017	2018	2019	2020
Human Papilloma Virus	151,903/342,223 (44.4)	19,173/44,906 (42.7)	18,713/43,802 (42.7)	16,003/40,800 (39.2)	17,430/41,366 (42.1)	16,332/38,771 (42.1)	16,707/37,960 (44.0)	21,645/46,393 (46.7)	25,900/48,225 (53.7)
*Haemophilus influenza* type B ^a^	221,759/254,603 (87.1)	25,443/29,159 (87.3)	26,877/30,462 (88.2)	28,120/31,521 (89.2)	27,832/31,429 (88.6)	28,146/31,845 (88.4)	28,434/32,878 (86.5)	27,863/33,561 (83.0)	29,044/33,748 (86.1)
Hepatitis A ^a^	201,204/253,036 (79.5)	26,211/28,997 (90.4)	24,361/27,030 (90.1)	23,518/26,176 (89.8)	22,801/25,528 (89.3)	23,581/30,827 (76.5)	28,443/43,009 (66.1)	27,332/38,028 (71.9)	24,957/33,441 (74.6)
Hepatitis B ^a^	159,808/200,208 (79.8)	18,269/23,600 (77.4)	19,282/24,711 (78.0)	19,966/25,026 (79.8)	19,565/24,754 (79.0)	20,108/25,402 (79.2)	20,766/25,759 (80.6)	20,675/25,475 (81.2)	21,177/25,481 (83.1)
Measles-Mumps-Rubella ^a^	189,653/239,229 (79.3)	22,380/30,228 (74.0)	22,690/30,050 (75.5)	24,238/30,186 (80.3)	23,600/29,853 (79.1)	23,868/30,123 (79.2)	24,517/30,505 (80.4)	24,075/29,447 (81.8)	24,285/28,837 (84.2)
Meningococcal ACY-135 ^a^	138,204/165,586 (83.5)	17,553/19,755 (88.9)	18,109/20,182 (89.7)	18,165/20,277 (89.6)	18,864/21,116 (89.3)	16,483/22,368 (73.7)	16,268/21,218 (76.7)	15,586/19,955 (78.1)	17,176/20,715 (82.9)
Pneumococcal Conjugate	218,373/244,963 (89.1)	25,374/28,217 (89.9)	26,313/28,874 (91.1)	27,443/29,990 (91.5)	27,216/30,066 (90.5)	27,396/30,566 (89.6)	28,092/31,649 (88.8)	27,689/32,581 (85.0)	28,850/33,020 (87.4)
Poliovirus ^a^	257,419/307,454 (83.7)	28,888/35,608 (81.1)	31,953/38,618 (82.7)	32,656/39,199 (83.3)	32,577/39,394 (82.7)	33,564/40,245 (83.4)	34,675/40,766 (85.1)	31,094/36,741 (84.6)	32,012/36,883 (86.8)
Rotavirus	141,292/147,063 (96.1)	16,054/16,605 (96.7)	17,143/17,784 (96.4)	17,875/18,465 (96.8)	17,662/18,404 (96.0)	17,481/18,255 (95.8)	18,191/19,025 (95.6)	18,206/18,980 (95.9)	18,680/19,545 (95.6)
Tetanus ^a^	401,957/456,120 (88.1)	47,437/54,635 (86.8)	49,674/56,465 (88.0)	50,154/56,893 (88.2)	50,747/57,921 (87.6)	50,775/58,024 (87.5)	51,850/58,696 (88.3)	49,182/55,724 (88.3)	52,138/57,762 (90.3)
Varicella ^a^	190,894/244,234 (78.2)	23,231/31,411 (74.0)	23,332/30,841 (75.7)	23,737/30,677 (77.4)	23,775/30,850 (77.1)	23,991/30,656 (78.3)	24,712/30,902 (80.0)	23,772/29,607 (80.3)	24,344/29,290 (83.1)

Vaccine acceptance is defined as vaccine administration over all offerings in the given year for the given antigen. ^a^ Antigens are aggregated across all vaccine product types containing the given antigen (i.e., monovalent and combination products).

**Table 6:** Vaccination Coverage of Routinely Recommended Advisory Committee on Immunization Practices Recommended Adolescent Vaccines in the Children’s Hospital of Philadelphia Primary Care Outpatient Network, 2013–2020.

Year	All Sites, Fully Vaccinated Adolescents (%)	Academic Urban Sites, Fully Vaccinated Adolescents (%)	Non-Academic Urban Sites, Fully Vaccinated Adolescents (%)	Suburban Sites, Fully Vaccinated Adolescents (%)
2013	3318/7940 (41.8)	717/1094 (65.5)	455/935 (48.7)	2146/5911 (36.3)
2014	4084/8325 (49.1)	994/1306 (76.1)	512/875 (58.5)	2578/6144 (42.0)
2015	4049/8076 (50.1)	956/1236 (77.3)	519/868 (59.8)	2574/5972 (43.1)
2016	4392/8650 (50.8)	1067/1314 (81.2)	517/864 (59.8)	2808/6472 (43.4)
2017	4497/8923 (50.4)	1113/1405 (79.2)	544/869 (62.6)	2840/6649 (42.7)
2018	4851/8814 (55.0)	1133/1371 (82.6)	672/974 (69.0)	3046/6469 (47.1)
2019	4408/8034 (54.9)	978/1255 (77.9)	536/835 (64.2)	2894/5944 (48.7)
2020	4658/7533 (61.8)	864/1048 (82.4)	446/611 (73.0)	3348/5874 (57.0)

Calculated by the specifications for the HEDIS^®^ Immunization for Adolescents (IMA) measure which includes one dose of meningococcal vaccine, one tetanus, diphtheria, acellular pertussis vaccine, and the complete human papillomavirus (HPV) vaccine series by an adolescents’ 13th birthday. For the tetanus, diphtheria, acellular pertussis (Tdap) vaccine component of the measure the tetanus (Td vaccine) was also counted for Tdap. NB: A CHOP quality improvement initiative began in 2019 to increase completion of HPV vaccination.

## Data Availability

Deidentified aggregate data can be made available upon request of the authors.
